# Improved WTCN-Informer Model Based on Frequency-Enhanced Channel Attention Mechanism and Wavelet Convolution: Prediction of Remaining Service Life of Ion Etcher Cooling System

**DOI:** 10.3390/s25164883

**Published:** 2025-08-08

**Authors:** Tingyu Ma, Jiaqi Liu, Panfeng Xu, Yan Song, Xiaoping Bai

**Affiliations:** School of Physics, Liaoning University, Chongshan Campus, Shenyang 110031, China; mty20212113@163.com (T.M.); liujiaqi_0606@163.com (J.L.); xupanfeng@lnu.edu.cn (P.X.)

**Keywords:** channeling attention mechanisms, DCT, etching machine cooling system, informer, remaining useful life, TCN, wavelet convolution

## Abstract

Etching has become a critical step in semiconductor wafer fabrication, and its importance in semiconductor manufacturing highlights the fact that it directly determines the ability of the fab to produce high-process products, as well as the application performance of the chip. While the health of the etcher is a concern, especially for the cooling system, accurately predicting the remaining useful life (RUL) of the etcher cooling system is a critical task. Predictive maintenance (PDM) can be used to monitor the basic condition of the equipment by learning from historical data, and it can help solve the task of RUL prediction. In this paper, we propose the FECAM-WTCN-Informer model, which first obtains a new WTCN structure by inserting wavelet convolution into the TCN, and then combines the discrete cosine transform (DCT) and channel attention mechanism into the temporal neural network (TCN). Multidimensional feature extraction of time series data can be realized, and the processed features are input into the Informer network for prediction. Experimental results show that the method is significantly more accurate in terms of overall prediction performance (MSE, RMSE, and MAE), compared with other state-of-the-art methods, and is suitable for solving the problem of predictive maintenance of etching machine cooling systems.

## 1. Introduction

The increasing complexity and specialization of modern manufacturing machines has resulted in significant investment costs, leading to a notable rise in the cost of machine hours. Consequently, there is a clear need to minimize machine downtime due to maintenance intervals and unexpected breakdowns. In the event of a component failure, the entire production line, or even another company dependent on the produced workpieces, may become idle. Therefore, in order to minimize the cost of such breakdowns, it is essential to identify them in advance and implement the appropriate measures.

The advent of artificial intelligence has given rise to a new approach to predictive maintenance, based on data-driven methodologies [1]. This has emerged as the most effective solution to the problems inherent in smart manufacturing and the management of industrial big data, particularly in the context of health monitoring (for example, fault diagnosis and remaining life assessment). The objective of predictive maintenance is to organize maintenance operations on the basis of the actual health status of the system, with the aim of indicating with greater precision the necessity for maintenance interventions. This is accomplished through the utilization of specialized models and techniques that are capable of diagnosing and predicting intricate system health conditions.

The impact of predictive maintenance in manufacturing is significant [2]. It has the potential to reduce maintenance costs by 25% to 35%, eliminate 70% to 75% of failures, reduce downtime by 35% to 45%, and increase production by 25% to 35%. These improvements can ultimately lead to a 15% to 60% increase in revenue. This is an attractive proposition for any manufacturing industry, particularly the semiconductor manufacturing industry, where equipment is costly, operations are intricate, and production conditions are complex [3]. In a wafer fabrication facility, a wafer is a silicon chip used to manufacture integrated circuits or photovoltaic products. The rapid development of the integrated circuit industry has resulted in an increased complexity in the processing of wafers. Among these techniques, ion etching technology is capable of processing the surface structure of products at the micron level. The ion mill etching (IME) machine [4] is one of the most utilized and effective machines for semiconductor etching technology. The utilization of the IME machine has become indispensable in the field of wafer processing. To prevent overheating, the IME machine employs a helium water system, designated as Flowcool, to reduce the temperature of the wafer. However, this specific system is prone to malfunctions, which can result in unanticipated periods of downtime and elevated maintenance expenses. It is therefore essential to implement a predictive maintenance strategy for ion mill etching machines in order to guarantee prompt maintenance and optimal operational efficiency.

**Figure 1:**
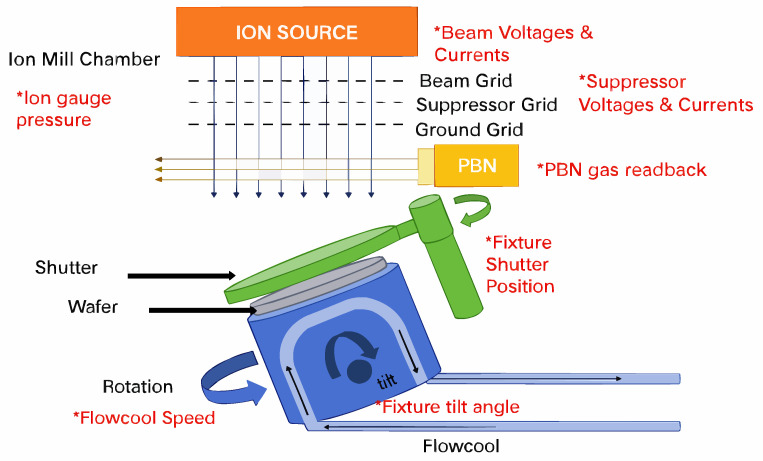
Ion mill etching machine and related parameters [6].

A complete IME process [5] is shown in Figure 1.

The Flowcool cooling system plays a pivotal role in the etching process. The system maintains the temperature of the wafer below the threshold level, thereby preventing damage to the surface of the wafer caused by overheating [7]. However, the system is susceptible to a multitude of failures, including gas leakage, component damage, and fluctuations in pressure. Such malfunctions have the potential to significantly impact the ultimate quality of the wafer product. In the context of these faults, preventive maintenance represents a common maintenance strategy. However, the efficacy of this strategy is not satisfactory. The selection of appropriate maintenance intervals represents a significant challenge. If the time interval is too short, healthy components may be replaced unnecessarily, resulting in redundant losses and inefficient use of resources. Furthermore, the implementation of longer maintenance intervals may result in inadequate maintenance procedures.

In order to address these issues, a number of prognostic techniques have been put forth for consideration. The techniques assess the condition of the equipment and forecasts its impending failure. The time to failure is also referred to as the remaining useful life (RUL) of the equipment [8]. Based on remaining useful life (RUL) predictions, effective maintenance measures can be implemented. Therefore, RUL prediction for flow cooling systems represents an effective predictive maintenance strategy. Predictions are made by detecting the condition of a component by collecting data (e.g., vibration signals are collected from monitoring sensors, which employ one of three approaches to prediction: model-based (or physically based), data-driven, or hybrid [9].

Model-based approaches include the development of mathematical or physical models [10,11] based on historical data to identify trends in component health states and may include statistical and computationally intelligent approaches. The process of IME requires complex interactions between a variety of particles and surfaces, and the dynamics are influenced by the unique properties of materials and process parameters. Therefore, the creation of physically based models to characterize degradation patterns, mechanisms, and failure modes is a major challenge [12]. However, this approach is time-consuming, costly, and requires extensive a priori knowledge. Despite its high accuracy, it is gradually falling out of favor.

In contrast, data-driven approaches primarily construct models based on historical data, subsequently identifying patterns associated with component health states. This encompasses, but is not limited to, machine learning (ML) methodologies [8]. Additionally, data-driven approaches can indirectly predict remaining useful life (RUL), estimate the degradation of components prior to failure, and predict information directly from the analysis of equipment signals. In general, the steps associated with data-driven approaches are divided into four parts, as outlined in [13]. The aforementioned steps, which include data acquisition, feature extraction, model construction and training, and RUL prediction and evaluation, do not necessitate a high level of expertise or complex equipment in comparison to model-based approaches. Furthermore, the rapid development of sensors and the field of big data have contributed to the increasing use of data-driven approaches.

In recent years, there has been an increasing prevalence of a combination of physics-based and data-driven system models. This method utilizes physics-based models to generate data regarding predictive parameters, which can subsequently be employed to synthesize an overall predictive model. This approach synthesizes the benefits of the aforementioned methods, yielding more precise predictions and a substantial reduction in training time. This method has already demonstrated outstanding performance in reducing wear [14], various types of rolling bearing failures [15,16], fatigue life [17], and other aspects. However, its utilization remains limited in industrial settings characterized by intricate processing operations.

Despite the increasing number of data-driven models, challenges remain in using these models for prediction in PHM. Kulbir Singh et al. [18] created a library of degradation curves based on sensor data in the Flowcool system and used Dynamic Time Warping (DTW) matching to predict the remaining useful life (RUL). This approach necessitates the storage of all training data, resulting in a linear increase in storage space and test time with the amount of training data. Furthermore, the accuracy of the prediction results is inadequate. In He and Jin [19] and Gupta, Huang, and Khosagani [20], the authors opted to compute the remaining useful life (RUL) using a trained long short-term memory (LSTM) network model. Additionally, to identify the early degradation time that triggers the prediction of the RUL, an early fault detection was employed prior to the estimation of the RUL [6]. However, the prediction accuracy was not satisfactory, primarily due to the failure to remove superfluous data through preprocessing. This paper presents a novel FECAM-WTCN-Informer model, which is developed based on a comprehensive analysis of the advantages and disadvantages of the previous models. The main contributions of this paper are as follows:

(1) The original TCN structure is optimized by replacing and adding the wavelet convolutional layer [21] to the TCN structure to obtain the WTCN structure. This new structure enhances the global sensory field of the data and improves the model’s ability to perform time series modeling.
(2) By integrating the discrete cosine transform (DCT) and channel attention mechanism into the WTCN, it is possible to achieve multi-dimensional feature extraction of time series data. The features resulting from the DCT, channel attention mechanism, and WTCN can then be inputted into the Informer network, thereby enhancing the accuracy and efficiency of prediction.
(3) In this paper, the optimization algorithm is used to train the network end-to-end, enabling the model to adaptively learn and identify the optimal hyperparameters, thereby enhancing the accuracy of prediction while reducing the bias error in prediction.

The remainder of this paper is organized as follows: Section 2 provides an overview of recent developments in prediction methods, with a focus on machine learning and deep learning approaches. Section 3 outlines the proposed model and its constituent components. Section 4 presents the predictions and validation results of the model. Finally, Section 5 offers a synthesis of the paper’s key findings and conclusions.

## 2. Related Works

Data-driven prediction methods based on machine learning (ML) and deep learning (DL), which belong to the domain of artificial intelligence, obviate the need for the construction of complex physical models. Instead, they employ historical data from sensors to extract features, construct models, and train them to predict the remaining useful life (RUL).

### 2.1. Machine Learning-Based Prediction Methods

ML methods can utilize a substantial quantity of data from sensors, historical maintenance records, and parameters [6]. These features assist dwelling algorithms in learning the relationship between numerous variables that affect the deterioration of the equipment’s health state. Sigaud et al. [22] employed an artificial neural network to predict RUL. This method detects models trained on data to make predictions. Theoretically, a multi-layer perceptron (MLP) with a single hidden layer can fit any continuous function. However, when faced with a sequential problem, it can only map inputs to outputs and is unable to model the variation in the sequence. Berghout et al. [23] provided a detailed account of the essential steps involved in utilizing machine learning techniques for RUL prediction. Additionally, they conducted an in-depth analysis of the prospective technological advancements and challenges that will shape the future of this field. Zhang et al. [24] employed Relevance Vector Machines (RVMs) in conjunction with Differential Algorithms (DE) to predict RUL using denoised data generated by wavelet algorithms. Mejia et al. [25] employed the Wavelet Packet Decomposition technique to extract coefficients from the raw sensor data and additionally utilized Gaussian Hidden Markov Models (HMMs) to evaluate the current health status of the device. This combination was used to assess the current health state of the device and predict the remaining useful life (RUL). The majority of these machine learning techniques necessitate the construction of features by humans, which consequently constrains the models’ capacity to process the data and generalize it for analysis.

### 2.2. Deep Learning-Based Prediction Methods

Deep learning represents a significant branch of machine learning that employs artificial neural networks and multi-layer data processing to emulate the cognitive functions of the human brain. Deep learning algorithms do not require feature engineering, as they are capable of autonomously extracting feature representations. Deep learning is distinguished by its more complex network architecture, which enables the extraction of degraded features from historical sensor data of monitored devices [26]. Additionally, deep learning has demonstrated superior efficiency in processing high-dimensional and unorganized data types, such as images and time series data. It can therefore be concluded that in the field of predictive maintenance, deep learning models will outperform other machine learning techniques [27].

#### 2.2.1. Convolutional Neural Networks and Improved Models

Convolutional neural networks (CNNs) represent a class of deep feedforward neural networks centered on convolution and pooling operations. Initially developed to address the challenge of image recognition in the field of computer vision, CNNs have also demonstrated robust feature extraction capabilities in predicting remaining useful life (RUL). This is attributed to their expanded receptive fields and efficient computational process [28]. A convolutional neural network (CNN) is a type of deep learning model that extracts local features from the input data and merges these features through a series of layered convolutional and pooling layers to generate high-level features. The ability to use convolutional kernels allows the model to sense changes over time in historical data and make predictions based on these changes [29,30]. The tuning required to enhance predictive maintenance typically involves the process of converting the input kernel from a two-dimensional filter to a one-dimensional filter. This process is designed to handle numerical data in time series datasets. Nevertheless, to enhance pattern recognition, the deployment of supplementary filters is necessary to discern more nuanced patterns [31].

Borovykh [32] and others enhanced the WaveNet–CNN model, which was inspired by the WaveNet speech sequence generation model, by utilizing the ReLU activation function and implementing parameterized skip connections. As datasets grow in size, convolutional neural networks (CNNs) tend to perform less effectively. In 2017, Dong et al. [33] proposed a solution in the form of the K-means–CNN model, which combines the capabilities of CNNs with those of the K-means clustering algorithm. This approach involves training the model to cluster similar samples in a large dataset into multiple smaller samples. The resulting model demonstrated promising performance on the millions of large-scale power loads datasets.

In 2018, Bai et al. [34] proposed a temporal convolutional network (TCN) architecture that required less memory and could be run in parallel. TCN introduced causal convolution, which ensured that future information would not be acquired in advance during training, and its backpropagation paths were different from the time direction to avoid the gradient vanishing and gradient explosion problems. To address the issue of information loss resulting from the inclusion of an excessive number of layers in a CNN, TCN incorporates residual connections, enabling the transfer of information across layers within the network.

The predictive efficacy of CNN is no longer superior to that of alternative network architectures, such as recurrent neural networks. Furthermore, while long-step-size time series prediction is a challenging problem, it is often used as a powerful module to connect to other advanced algorithmic models for prediction tasks.

#### 2.2.2. Recurrent Neural Networks and Improved Models

Recurrent neural networks (RNNs) are deep learning models for learning temporal dimension features. They were first proposed by Jordan [35] in 1990. RNN units are connected together in long chains recursively in the direction of sequence development. The input to the model is the sequence data. The RNN leverages the stored knowledge within the hidden units, which pertain to past sequence observations, to examine temporal relationships within the input sequence at a specific point in time. Catelani et al. [36] integrated RNN with filtering-based RUL prediction techniques. The objective of this method is to enhance the efficacy of RNN through the utilization of a Genetic Algorithm (GA). The employment of gradient-based backpropagation for the modification of network weights presents two potential limitations to RNN. As the number of hidden layers increases, the gradient explosion or gradient decay problem arises, particularly in the context of long-term prediction. This results in an RNN that is only capable of capturing short-term patterns, effectively limiting its memory.

In order to address the shortcomings of RNN models, Hochreiter [37] proposed the long short-term memory (LSTM) model in 1997. The LSTM incorporates a gating mechanism to address the limitations of traditional RNNs in managing memory retention and forgetting processes. Jianjing et al. [38] introduced an LSTM neural network that incorporates state units to retain long-term memory. Gated Recurrent Units (GRUs) are analogous to LSTMs in that they employ gating mechanisms to regulate long-term dependencies. However, the primary distinction between them pertains to the specific gates utilized. Chen et al. [39] proposed a two-stage methodology comprising Kernel Principal Component Analysis (KPCA) for feature extraction and a GRU-based architecture for predicting remaining useful life (RUL).

The GRU (Gated Recurrent Unit) is a recurrent neural network that offers slight improvements over the LSTM, as proposed by Cho et al. (2014) [40]. The GRU model combines the forgetting gate and input gate into a single “update gate,” as well as combining the cell state and hidden state. In comparison to the standard LSTM model, the GRU model is simpler and is gaining more and more attention and applications. The GRU network represents a successful variant of the LSTM structure. Furthermore, GRU networks can dynamically control the forgetting behavior of different units, such as LSTM networks, but are simpler in structure. GRU networks have been widely used in various fields, including forecasting and health management. For instance, they have been employed in fault diagnosis of hydroelectric generator sets [41], forecasting of aviation propulsion systems, machine health monitoring [42], and other applications.

In 1997, Schuster et al. [43] extended the regular recurrent neural network (RNN) to bidirectional recurrent neural networks (Bi-RNNs). Unlike the RNN, which processes information in only one direction, the Bi-RNN can process input information without restriction up to predefined future frames by simultaneously training in both the forward and backward directions. It is possible to obtain information regarding both the past and the future simultaneously. In regression prediction experiments on artificial data, the training time for Bi-RNNs is approximately the same as that for RNNs, yet the former achieves superior prediction results.

In 2005, Graves et al. [44] proposed a bidirectional long short-term memory (Bi-LSTM) structure analogous to Bi-RNN, comprising two independent LSTMs spliced together. Wang et al. [45] introduced a bidirectional LSTM model in which the attention mechanism is employed for the RUL regression of LSTMs.

Recurrent neural network (RNN) methods can capture and utilize both long-term and short-term time dependencies for prediction purposes. RNN-based methods have been successfully applied to a wide range of prediction problems, including the prediction of machine wear, turbofan engine runout (RUs), centrifugal compressor performance estimation, and electrical and bearing health state estimation.

#### 2.2.3. Transformer Class Model

Transformer [46] represents a novel deep learning framework that diverges from the conventional CNN and RNN architectures. The fundamental component of Transformer is the self-attention module, which can be conceptualized as a fully connected layer whose weights are dynamically generated based on the pairwise similarity of input patterns. The framework’s minimal number of parameters and reduced computational requirements under comparable conditions render it well-suited for modeling long-term dependencies. The exceptional capacity of Transformer to capture long-term dependencies and interactions is highly advantageous for time series modeling tasks, and it can demonstrate high performance in a variety of time series tasks.

The Transformer model addresses the challenge of predicting a long sequence through a multiple prediction approach. Building upon the classical Transformer encoder–decoder structure, BUAA proposes the Informer [47] model to address the limitations of the Transformer class of deep learning models in predicting long sequences. It is capable of generating the desired long sequence results in a single step.

In 2021, Alibaba proposed Aliformer [48], which is based on a bidirectional Transformer. This was done in order to solve the problem of accurate time series sales prediction in e-commerce. This utilizes historical information, current factors, and future knowledge in order to predict future values. The self-attention layer in Aliformer uses the consistency of a priori knowledge in order to guide the transmission of time series information. It also proposes a training strategy that emphasizes the future. This makes the model more focused on the use of future knowledge. Wu [49] proposes the Autoformer architecture for long-term time series prediction, introducing an autocorrelation mechanism to replace the self-attention mechanism. For sequences of length L, this mechanism achieves a computational complexity of O(LlogL) and breaks the information usage bottleneck. To more effectively capture the global features of time series, the FEDformer (frequency-enhanced decomposed Transformer) [50], proposed in 2022, applies Transformer to the frequency domain. It employs two attention modules to address the application of attention in the frequency domain using Fourier transform and wavelet transform, respectively. In 2023, a Conformer [51] model for multivariate long-periodic time series prediction was proposed to address the efficiency and stability issues associated with long sequence prediction tasks with significant periodicity. The proposed model incorporates several optimizations, including Fourier variation, multi-frequency sequence sampling, and normalized flow, to generate time series in a generative manner with high noise immunity.

Transformer-like algorithms are currently employed in a multitude of tasks within the domain of artificial intelligence, particularly in the context of time series prediction. The application areas encompass, but are not limited to, the prediction of remaining useful life (RUL) of industrial bearings, aircraft engines, robotic gearboxes, and financial stocks, among others. The construction of a model based on Transformer can overcome the limitations of previous algorithms, simultaneously exhibiting proficiency in capturing both short- and long-term dependencies, effectively addressing the challenge of long series prediction and enabling parallel processing.

## 3. Methodology

### 3.1. Overall RUL Forecasting Program

**Figure 2:**
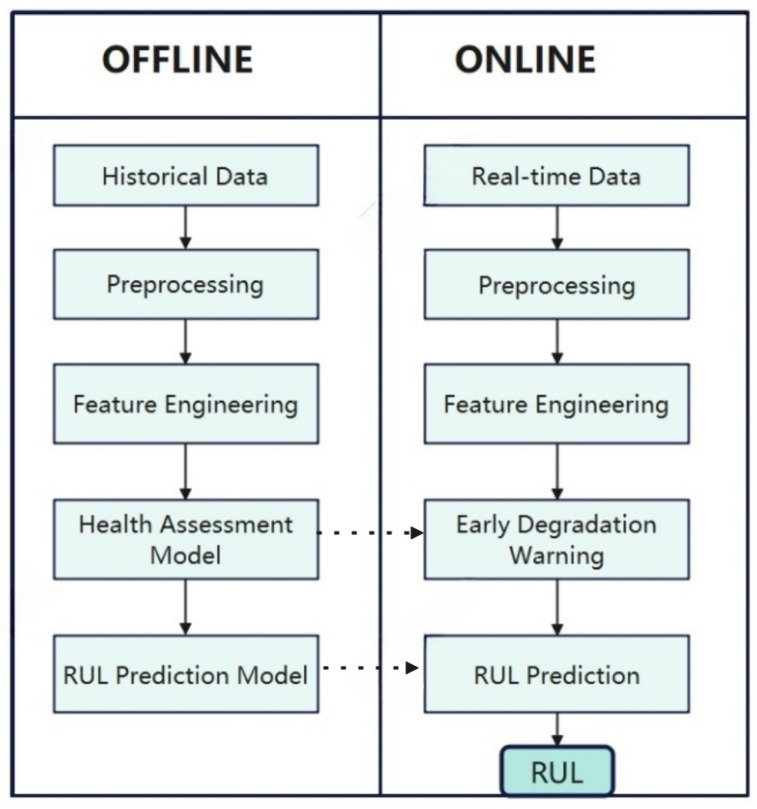
RUL prediction procedure.

The complete prediction program is illustrated in Figure 2. A comprehensive RUL prediction process comprises two principal components: offline modeling and online application. In the offline modeling phase, the prediction model is constructed through the comprehensive utilization of historical monitoring data or physical models of subsystem components. During the online phase, the requisite preprocessing of real-time sensor data and environmental parameters can be conducted. The aforementioned data are employed for the real-time prediction of remaining useful life (RUL) based on the modeling conducted in the offline phase.

This study divides the dataset into a training set and a test set. The training set is used for model construction, while the test set is used to evaluate model performance and validate online prediction capabilities. The construction of offline models includes data preprocessing, feature engineering, degradation warning health assessment models, and remaining life prediction models. Data preprocessing primarily involves data filtering, removal of redundant data, imputation of missing data, standardization, and normalization, aiming to improve data quality, reduce data dimensions, optimize data distribution and consistency, and thereby enhance algorithm efficiency. Feature engineering focuses on extracting more representative features through data collection, cleaning, transformation, and selection, providing the optimal feature set to enhance the model’s predictive accuracy and generalization capabilities.

Additionally, it simplifies the computational process. The following three subsections will present the components of the degradation warning model, the RUL predictive model, and the overall model.

### 3.2. Degradation Early Warning Health Assessment Model

**Figure 3:**
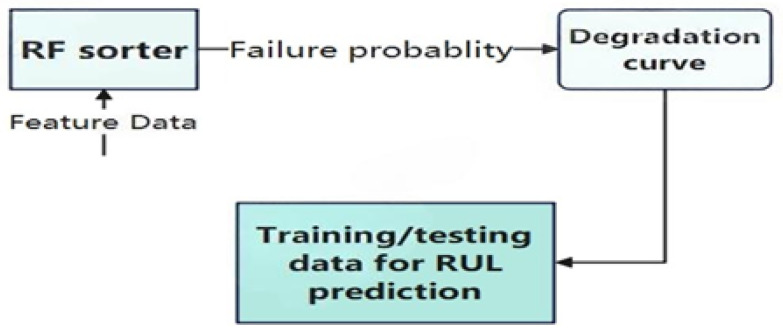
Degradation warning procedure.

Health assessment is done to show the operational status of the system and the damage that has occurred. Health assessment plays a critical role throughout the entire system, as it can accurately reflect the operating status of equipment or systems in a timely manner, helping to prevent potential failures, extend equipment life, reduce maintenance costs, and improve overall safety and reliability. In this paper, the health assessment model is trained to obtain degradation curves and the degradation curves are used to determine the early degradation warning points while triggering the RUL prediction. The early degradation warning procedure is shown in Figure 3.

**Figure 4:**
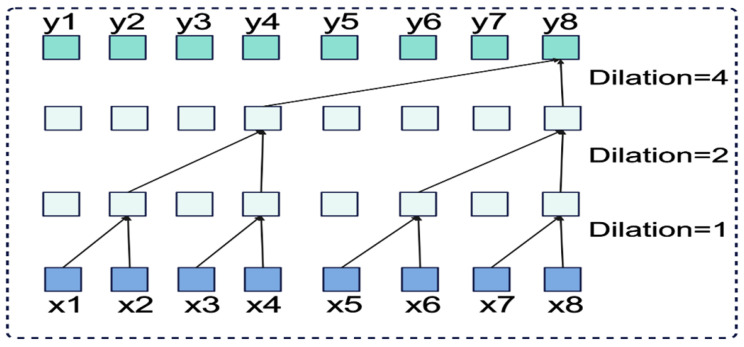
Dilation causal convolution operation.

The binary classification model in Figure 4 is constructed by learning the relationship between the processed features and the operational status labels. Health assessment uses a random forest classifier, which is an ensemble model composed of multiple decision trees. The model uses bootstrapping to repeatedly sample from the original data, generating multiple training sets, each of which is used to train a decision tree. When constructing the decision tree, a portion of the features is randomly selected at each node, and the feature with the highest information gain is selected for splitting until no further splitting is possible. The process ends after it cannot continue splitting and generates multiple decision trees in this manner to form a random forest. Each tree makes a prediction on the new data, and the final output of the forest is the average of the predictions of the trees. About 37% of the samples are not selected due to put-back sampling (probability = ${\left(1-1/N\right)}^{N}\approx 0.368$), and these out-of-bag (OOB) data can be used for model evaluation. Compared to single decision trees, random forests are more resistant to interference due to the introduction of randomness, effectively mitigating overfitting and providing better performance.

Degradation warning health assessment models are of paramount importance in situations where it is not feasible to predict RUL when the system in question shows no indications of failure. The utilization of data obtained from a system operating within normal parameters is an ineffective approach for training purposes and also results in a reduction in prediction accuracy. Models that have not been trained to recognize degradation patterns are unable to make accurate predictions.

### 3.3. FECAM-WTCN-Informer Model Component

#### 3.3.1. Modified Temporal Convolution Network (TCN)

Sequence modeling traditionally relies on recurrent neural networks [34], but simple convolutional structures actually outperform recurrent models like LSTMs on various tasks while offering better long-term memory.

Temporal Convolutional Networks (TCNs) demonstrate superior memory retention compared to conventional RNNs, including LSTM and GRU variants. TCNs not only provide extended effective memory spans but also deliver higher accuracy with simpler, more interpretable designs.

These findings challenge the conventional association between sequence modeling and recurrent architectures, suggesting TCN-based approaches may be more optimal for sequential data analysis in deep learning applications.

TCNs effectively adapt convolutional neural networks for sequence modeling through a simplified architecture. The key difference from standard convolutional neural networks (CNNs) lies in the implementation of causal convolutions. The key features of TCNs are as follows:

(1) As shown in Figure 4, dilated causal convolution achieves larger receptive fields than standard convolution with the same kernel size. Unlike regular convolution, causal convolution only uses past information, maintaining temporal order. The receptive field size depends on network depth, filter dimensions, and dilation factors. This design prevents future information from affecting past predictions, ensuring proper temporal causality.

(2) Similar to RNNs, TCNs process variable-length input sequences and generate corresponding output sequences of equal length. To handle long-term dependencies, TCNs employ dilated convolution, which achieves exponentially expanding receptive fields by introducing fixed intervals between adjacent filter elements and incorporating past information direction indicators.

For a one-dimensional input vector $x\in R^{n}$ and filter function $f:\left\{0,1,\cdots ,k-1\right\}\to R$, the dilated convolution operation D applied to sequence element s is mathematically expressed as

(1) $$D\left(S\right)=\left(X*f_{d}\left(s\right)\right)=\sum_{i=0}^{k-1}f\left(i\right)x_{s-d\cdot i}$$

where *d* denotes the dilation factor and k represents the filter size. The TCN’s receptive field expands proportionally with the dilation factor, achieving an effective historical span of (k−1)d for each layer.

Additionally, TCNs adopt generalized residual modules instead of standard convolutional layers to maintain stability in increasingly deep and complex networks. Following He et al.’s approach [19], these residual modules allow layers to learn identity mapping modifications rather than complete transformations. The identity mapping combines the transformation output F with the block’s input x:

(2) $$y=F\left(x,\left\{Wi\right\}\right)+x$$

Here, *x* and *y* denote the input and output vectors of the respective layer. The function $y=F\left(x,\left\{Wi\right\}\right)$ represents the residual mapping for learning, which facilitates improved performance in deep network architectures.

(3) TCN employs a combination of exceedingly deep networks (with residual structures) and dilated convolutions to construct exceptionally long effective history sizes (i.e., the capacity of the network to examine a vast temporal span to make predictions).

**Figure 5:**
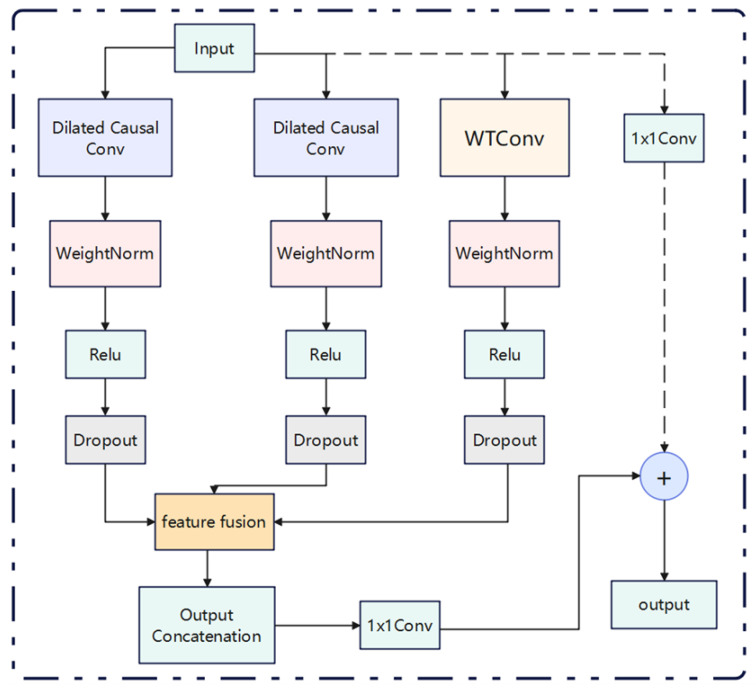
TCN residual blocks with parallel structure.

Figure 5 illustrates the modified TCN residual block, which comprises two parallel layers of dilated causal convolution and one layer of wavelet convolution [21].

The authors of the WTConv layer employed the Haar wavelet transform, a straightforward and effective wavelet substrate. The WTConv layer is a layer that utilizes a cascaded wavelet decomposition and performs the convolution of a set of small convolutional kernels, each focusing on a distinct frequency band of the input with progressively larger sense fields. This process is capable of assigning greater significance to low-frequency information in the input while introducing a minimal number of trainable parameters. Although the authors have previously applied this layer to image problems in computer vision, in this paper it is downscaled and applied to time series problems by combining it with a TCN structure to obtain an improved WTCN module.

The two branches on the left side of the image are causal convolutions with different expansion rates. These convolutions are used to capture the dependencies of different time scales. The WTConv branch is enhanced by wavelet transform convolution. This convolution is used to extract the frequency domain features. This process breaks through the traditional time domain limitation. It also maintains the causality. Furthermore, it is suitable for real-time applications. Finally, it has a perfect mechanism for multidomain feature fusion.

Specifically, after the input data, in addition to entering the two layers of the original expansion causal convolution, it will also go through the WTConv layer to extract features at different frequencies. Subsequently, it is added to the feature fusion layer with the original TCN, before the location of the combined features. The fused features are then connected to the residuals of the remaining modular features through a one-dimensional convolution. To enhance the long-term memory capability of TCN, the expansion is combined with causal convolution in order to support autoregressive prediction. Furthermore, residual concatenation is employed to facilitate multi-layer stacking while preventing model overfitting. To guarantee the stability of the deep neural network, a residual block with shortcut connections is incorporated into the TCN. Furthermore, spatial culling and weight normalization are applied after each dilation causal convolution, which serves to mitigate the overfitting issue while simultaneously ensuring regularity and accelerating the training process, thereby enhancing the performance of the deep neural network. Moreover, the inputs and outputs of the residual block are integrated through the addition of alignment elements, which are combined through 1 × 1 convolutional layers.

#### 3.3.2. Frequency-Enhanced Channel Attention Mechanism

In the context of time series, frequency is an indispensable piece of information. Indeed, real-world datasets often contain a wealth of frequency data. The majority of mainstream frequency information extraction methods are based on the Fourier transform (FT). However, due to the Gibbs phenomenon, oscillatory approximation occurs on both sides of the sequence when the values on either side are markedly disparate, resulting in the introduction of high-frequency noise. To address this issue, a novel frequency-enhanced channel attention mechanism has been proposed. This mechanism adaptively models the frequency interdependence between channels based on the discrete cosine transform, thereby avoiding the high-frequency noise caused by the periodicity problem (i.e., the Gibbs phenomenon) during the Fourier transform process. The frequency-enhanced channel attention mechanism [50] module can be flexibly applied to different networks. This mechanism includes the discrete cosine transform (DCT) [52] and Squeeze-and-Excitation Network (SENet) [53].

Discrete cosine transforms (DCTs) are separable transforms whose transform kernels are cosine functions. In addition to their general orthogonal transform properties, the basis vectors of their transform arrays are well-suited for characterizing the correlation between human speech signals and image signals. The fundamental equation of the one-dimensional discrete cosine transform (1D DCT) is as follows:

(3) $$B_{l}^{i}=\cos\left(\frac{\pi l}{Ls}\left(i+\frac{1}{2}\right)\right)$$

And 1D DCT can be written as

(4) $$f_{1d}^{l}=\sum_{i=0}^{Ls-1}x_{i}^{1d}B_{l}^{i}$$

In Equation (4), $l\in \left\{0,1,2\cdots ,L_{s-1}\right\}$, $f_{1d}^{l}\in R^{l}$ is the 1D DCT frequency spectrum, $X^{1d}\in R^{l}$ is the input, and L is the length of $X^{1d}$. Accordingly, the inverse 1D DCT can be written as

(5) $$x_{1d}^{l}=\sum_{i=0}^{L_{S}-1}f_{l}^{1d}B_{l}^{i}$$

These include $i\in \left\{0,1,2\cdots ,L_{s-1}\right\}$, $f_{1d}^{l}\in R^{l}$.

**Figure 6:**
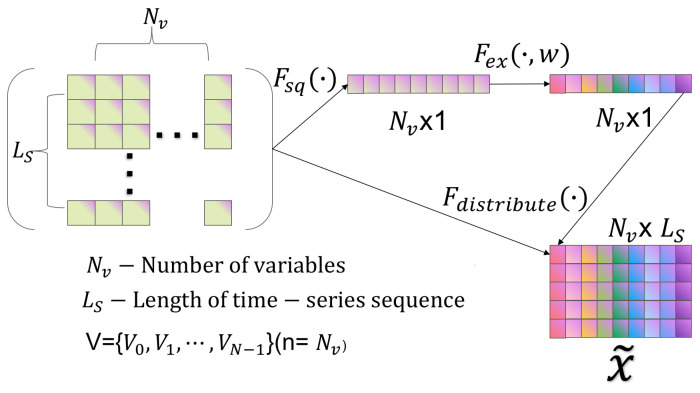
SENET channel attention (Squeeze-and-Excitation Network).

In recent years, there has been a notable increase in the use of attention mechanisms in deep neural networks. These mechanisms direct computational resources to the most informative part of the input signal, thereby greatly improving performance in a multitude of tasks. In this paper, a channel attention mechanism, termed SENet, is employed to enhance the expressive power of the network. SENet achieves dynamic adjustment of each channel feature by modeling the dependencies between channels, thus leveraging global information to reinforce meaningful features and suppress useless ones. Its configuration, illustrated in Figure 6, comprises two primary stages. Initially, the spatial dimensions of the input feature map are diminished via a compression phase, and, subsequently, the weights of each channel are determined in an excitation phase. In conclusion, the weights are applied to the input feature map in order to generate the enhanced output feature map.

The figure has been designed with a bypass branch in addition to the traditional convolution operation. Firstly, the compression operation (denoted by $F_{sq}\left(\cdot \right)$ in the figure) compresses the spatial dimensions of the features, converting each 2D feature map into a single number. This is similar to global pooling, but the number of channels remains unchanged. In the $F_{sq}\left(\cdot \right)$ model, the extraction of global information is undertaken, with subsequent allocation to each individual channel. Subsequently, the excitation operation ($F_{ex}\left(\cdot \right)$) generates the weights for each channel. The weight parameter w is learned through a two-layer fully connected network. This network first reduces the dimensionality, then restores the dimensionality, and finally goes through a Sigmoid function that captures the correlation between the channels. Subsequent to the computation of the weights, they are applied to the corresponding channels, thus adjusting the importance of each channel to the specific task.

#### 3.3.3. Informer

**Figure 7:**
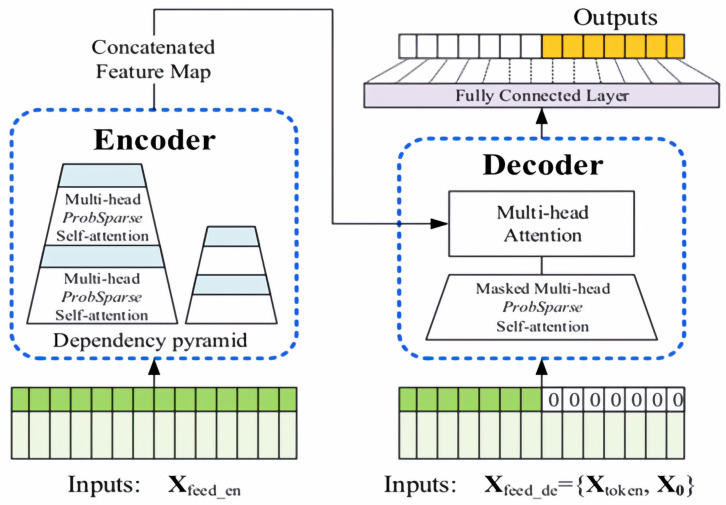
Informer model structure diagram.

In recent years, research on sequence prediction has primarily concentrated on the prediction of short sequences. As the length of input sequences increases, the computational complexity of traditional models rises concomitantly, resulting in a notable decline in prediction capability. Long sequence time series forecasting (LSTF) has emerged as a prominent area of research in sequence forecasting due to its extensive historical data, high computational complexity, and notable forecasting accuracy. However, despite these advances, long sequence forecasting has not yet reached a comparable level of success. LSTF necessitates a model with a high prediction capability, namely one that is capable of accurately capturing the long-term dependency relationship between the output and input. In comparison to RNN-type models, Transformer demonstrates considerable promise in the expression of long-distance dependencies. However, Transformer is not directly applicable to LSTF problems due to several significant limitations, including high memory usage and inherent constraints of the encoder–decoder architecture. The Informer model was developed based on the Transformer architecture, and its structural configuration is illustrated in Figure 7.

Figure 7 shows the specific implementation of the Informer architecture. The encoder part (left side) is specifically responsible for capturing long-range dependencies in long sequence inputs. Its workflow is as follows: after receiving long sequence inputs, it processes them through the ProbSparse self-attention module and self-attention distillation module and finally generates feature representations. The decoder component (right side) aims to predict the entire output of a long sequence through a single forward pass. The specific process is as follows: after receiving the long sequence input (with the target prediction portion set to zero), it interacts with the encoded features via a multi-head attention mechanism, ultimately predicting the target output portion in a single forward pass.

Compared to traditional Transformer models, Informer achieves three key innovations in its architectural design: the ProbSparse self-attention mechanism, self-attention distillation technology, and a generative decoder structure.

The ProbSparse self-attention mechanism reduces the time complexity of the attentional computation process. Unlike the classical self-attention mechanism query vector evaluation equation, which measures the sparsity of the query, Informer employs KL dispersion to compute the relative entropy of the probability distribution of attentions of the query with respect to the probability distribution of the uniform distribution. The evaluation formula for the sparsity of the ith query is:

(6) $$M\left(q_{i},k\right)=\ln\sum_{j=1}^{L_{K}}e^{\frac{q_{i}k_{j}^{T}}{\sqrt{d}}}-\frac{1}{L_{k}}\sum_{j=1}^{L_{K}}\frac{q_{i}k_{j}^{T}}{\sqrt{d}}$$

The initial term represents the Log-Sum-Exp (LSE) for all keys, while the second term denotes their arithmetic mean. Subsequently, the formula for ProbSparse self-attention is derived from Equation (6), as shown in Equation (7).

(7) $$A\left(Q,K,V\right)=\mathrm{Softmax}\left(\frac{\bar{Q}K^{T}}{\sqrt{d}}\right)V$$

In contrast to other approaches, ProbSparse self-attention requires the computation of a dot product operation for each query–key pair. Moreover, to circumvent the numerical stability issue associated with the Log-Sum-Exp (LSE) arising from the truncation of computational precision during the calculation process, the method in question performs the computation of upper bounds for sparse evaluation. This method provides a solution to the issue of Transformer’s secondary computational complexity.

The self-attention distillation mechanism can effectively reduce the time dimension of the feature map, thus reducing the memory consumption and solving the problem of high memory usage of Transformer. In comparison to the conventional step-by-step dynamic decoding approach, Informer employs the output of ProbAttention to calculate the final result. Subsequently, Full Attention is computed on the queries, keys, and values to retrieve the masked portion, enabling the model to predict all outputs directly through a forward process, circumventing the necessity for step-by-step dynamic decoding. The parallel generative decoder mechanism has the potential to markedly enhance the efficiency of long sequence prediction.

#### 3.3.4. Parameter Optimization Component

In order to further improve the accuracy and efficiency of the model, this paper uses the Sparrow Search Algorithm (SSA) to train the model end-to-end and thus optimize the hyperparameters of the model. The SSA algorithm was proposed in 2020 by Xue et al. [54]. The idea of the algorithm is inspired by the two foraging behaviors of sparrows in nature, and there are two types of sparrows: discoverers and joiners. The explorers are responsible for finding food and providing information about the foraging area to the population. Joiners use the foragers to obtain food. In their natural state, individuals monitor each other, and foragers in a sparrow population will typically compete for food resources from their high-foraging peers in order to increase their own foraging rate. While foraging, all individuals remain alert to their surroundings to prevent the arrival of predators. The algorithmic steps are as follows:

(1) The initial population and related parameters must be initialized, and the fitness value of the initial population must be calculated.

(2) The finder position must be updated according to Equation (8).

(8) $$X_{i,j}^{t+1}\left\{\begin{array}{cc} X_{i,j}^{t}\cdot \exp\left(\frac{-i}{\alpha \cdot iter_{\max}}\right) & \;\mathrm{if}\;R_{2}<ST\\ X_{i,j}^{t}+Q\cdot L & \;\mathrm{if}\;R_{2}>ST \end{array}\right.$$

When the warning value $R_{2}$ is less than the safety value ST, it indicates that the search range of the discoverer is relatively large. Conversely, when the warning value $R_{2}$ is equal to or greater than the safety value ST, it indicates that there are a certain number of predators and that it is necessary to relocate to a safer area.

(3) The position of the joiner should be updated in accordance with the specifications set forth in Equation (9).

(9) $$X_{i,j}^{t+1}=\left\{\begin{array}{cc} Q\cdot \exp\left(\frac{X_{worst}^{t}-X_{i,j}^{t}}{i^{2}}\right) & \;\mathrm{if}\;i>n/2\\ X_{p}^{t+1}+\left|X_{i,j}^{t}-X_{p}^{t+1}\right|\cdot A^{+}\cdot L & \;\mathrm{otherwise}\; \end{array}\right.$$

In the code implementation, the number of joiners, represented by the variable “*n*”, is updated by modifying the position of the last 80% of individuals, which is equivalent to updating the position of the joiner. In the event that the aforementioned joiner is identified as the superior joiner of the initial half, the position is updated in accordance with the first sub-equation. Conversely, in the event that the aforementioned joiner is identified as the inferior joiner of the subsequent half, it can be considered to be analogous to the sparrow being in a state of extreme hunger and requiring to fly to an alternative location at random.

(4) Update the position of the sparrow that is aware of the danger, as shown in Equation (10).

(10) $$X_{i,j}^{t+1}=\left\{\begin{array}{cc} X_{\mathrm{best}\;}^{t}+\beta \cdot \left|X_{i,j}^{t}-X_{\mathrm{best}\;}^{t}\right| & \;\mathrm{if}\;f_{i}>f_{g}\\ X_{i,j}^{t}+K\cdot \left(\frac{\left|X_{i,j}^{t}-X_{\mathrm{worst}\;}^{t}\right|}{\left(f_{i}-f_{w}\right)+\varepsilon }\right) & \;\mathrm{if}\;f_{i}=f_{g} \end{array}\right.$$

(5) The stop condition should be evaluated to ascertain whether it is satisfied. In the event that this condition is met, the optimal sparrow position should be output. Otherwise, the process should resume at step 2.

In this paper, the SSA algorithm is employed to optimize the selection of learning rate, number of neurons, and batch size among the parameters, thereby enhancing the fitting effect of the model and reducing the error.

### 3.4. FECAM-WTCN-Informer Model

**Figure 8:**
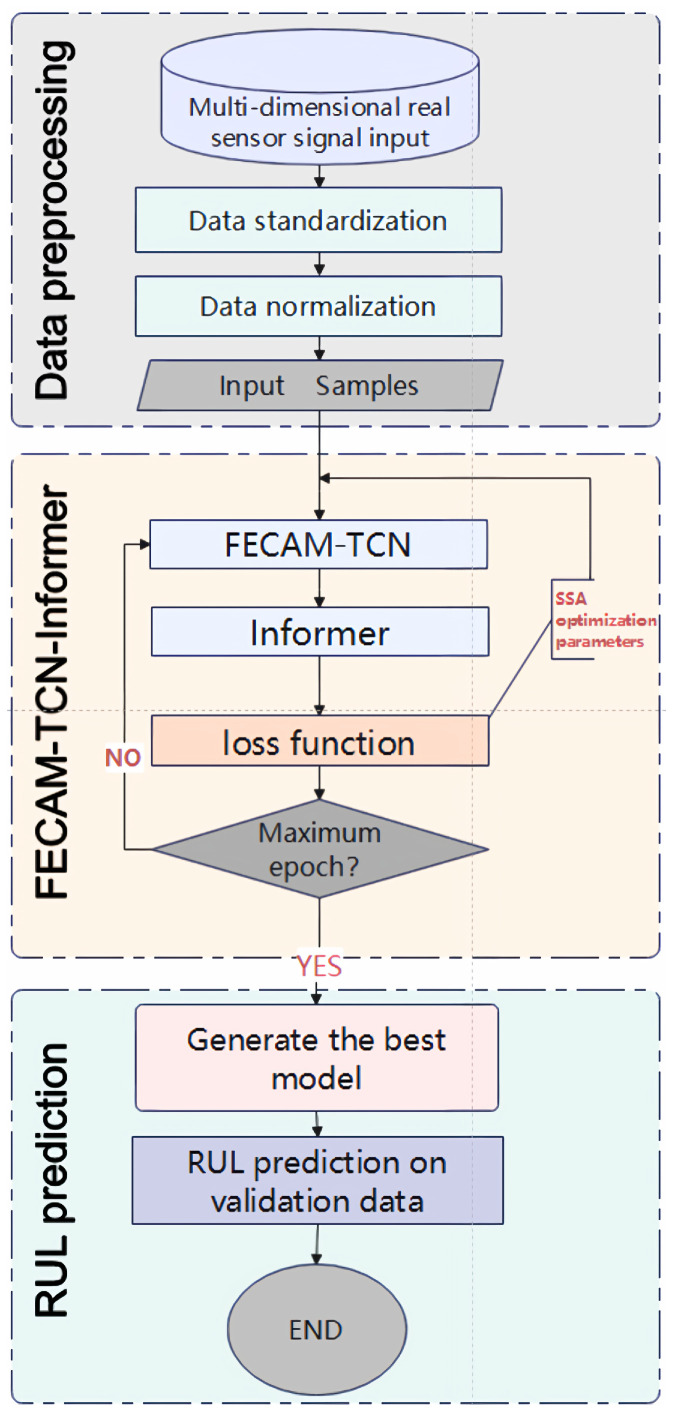
FECAM-WTCN-Informer flowchart.

The flowchart of the FECAM-WTCN-Informer model, as developed in this paper, is shown in Figure 8. The aforementioned components are described in greater detail in the preceding subsections. The FECAM-WTCN module is a time-domain convolutional network based on the frequency-enhanced channel attention mechanism. This, in conjunction with the Informer module and the parameter optimization module, constitutes the FECAM-WTCN-Informer model.

The multidimensional raw sensor signals are initially subjected to preprocessing, after which the samples are constructed using the sliding window method. Subsequently, the time series samples, comprising the actual sensor signals and labels, are fed into the FECAM-WTCN-Informer model. The feed-forward propagation enables the multi-dimensional feature extraction of the time series data using FECAM-WTCN-Informer. The processed features are then fed into the Informer network, which reinforces the key time points to improve the accuracy and efficiency of the prediction. This constitutes the entire process of an epoch. Once the termination conditions have been met, the epoch is terminated and the model is saved. Subsequently, the SSA algorithm is employed to optimize the key parameters based on the MSE parameters, namely the number of neurons, the learning rate, and the batch size. This is followed by a repetition of the training process, with the objective of obtaining the optimal model, which is then saved. The data to be validated is then input into the model for subsequent experiments and predictions in real-world applications.

## 4. Predictive Results of the Model

The present study evaluates the performance of the proposed FECAM-WTCN-Informer model for predicting the remaining useful life (RUL) of ion etching tools. This is based on the Flowcool dataset, and the study compares the model with existing state-of-the-art methods. The dataset and the data preprocessing methods are detailed in Section 4.1, and the performance evaluation metrics are presented in Section 4.2. In Section 4.3, the reader will find reports on the following:

- The experiments that were conducted to study the process of ablation;
- The results of the comparison experiments;
- The predictive performance of the model on the test set.

All experiments are conducted within a uniform environment, characterized by predefined configurations. These configurations encompass Python 3.9, PyTorch 2.0.0 + CU118, an Intel Xeon Silver 4210R CPU, and an NVIDIA RTX A5000 GPU.

### 4.1. Data Description and Preprocessing

#### 4.1.1. Flowcool Dataset

The Flowcool dataset utilized in this study was published in the 2018 PHM Data Challenge. The 2018 PHM Data Challenge dataset is a real-world dataset employed to investigate the failure behavior of multiple ion mill etching tools utilized in the IME process. The dataset encompasses 20 tools equipped with a diverse array of sensor data utilized for process monitoring, including 20 sensor measurements and operational parameters of the IME machines. The dataset comprises 24 feature variables, collected at 4-second intervals. These include five categorical features (e.g., wafer IDs, tool IDs, and recipes) and 19 numerical features (including voltages, currents, pressures, and flow rates). Table 1 provides a comprehensive overview of the dataset. The various run-to-failure cycles within each tool are integrated based on the type of failure observed. The ability to effectively predict potential failures allows for the proactive scheduling of equipment maintenance, thereby preventing unplanned downtime and maintaining overall equipment efficiency during the etch process. Additionally, the dataset encompasses the temporal occurrence of three distinct failure types: flow-cooling leaks (F1), flow-cooling pressure exceeding the threshold for pump monitoring (F2), and flow-cooling pressure below the limit (F3). Given the disparate operating and consumption states of the devices, it is essential to construct a model for each device independently. In this paper, we utilize the 01_M02 device as a case study for developing a model for predicting remaining useful life (RUL).

The dataset provides the time of failure, defined as the point at which the operator initiates the shutdown of the machine for maintenance purposes. This specific time point serves as the focal point for our predictive analysis. The ion mill etching process is characterized by three distinct types of failures. However, it should be noted that the dataset under scrutiny contains more failures than the ion mill etching process can produce. Furthermore, the dataset exhibits a significantly lower frequency of failures compared to standard data. A comparative analysis reveals that the F2 type of data experiences a reduced number of failures compared to the F1 and F3 types. This discrepancy in the dataset leads to a deviation in the effectiveness of the model for the F2 type. Therefore, it is necessary to augment the proportion of faulty data, as delineated in the preprocessing section below.

#### 4.1.2. Data Preprocessing

It is necessary to screen the data in order to remove any data that is not pertinent to the etching process, particularly data collected when the etching is not in progress. In the initial stage of data preprocessing, any data that is deemed to be unreasonable or absent is removed. This effectively reduces the noise encountered during model training and improves the performance of the test dataset. Given that the scales of the sensors in question vary, it is necessary to normalize the sensor values.

A comprehensive evaluation of the IME process and the configuration of the equipment has revealed that the position of the baffle is instrumental in ascertaining the operational status of IME equipment. When the baffle is closed, the wafer is isolated from the ion beam, thereby preventing the etching process from proceeding.

The fixed baffle position (FSP) parameter is employed to characterize the baffle status. This parameter is a categorical variable with five distinct categories: The set contains the numbers zero, one, two, three, and two hundred and fifty-five. When the fixed shutter position is designated as “0,” both the fluid cooling pressure and the ion pressure gauge pressure are observed to be at their minimum values. As the shutter position transitions from “0” to “1,” both pressure parameters exhibit a substantial increase. Upon attaining the preset pressure level, the fixed shutter position transitions to “1,” thereby signifying that the shutter is open and the fluid cooling subsystem is functioning optimally. When the shutter is closed, the fixed shutter position reverts to “0,” causing a precipitous drop in pressure.

Therefore, this study selected data with the fixed damper position parameter set to “1” to represent the operational state of the ion milling equipment.

According to the findings of the present study, the remaining useful life (RUL) can be determined through the implementation of a 4-s data sampling interval. The RUL can be quantified by the number of samples from the current time to the time of failure. According to official data, the actual time of failure may be significantly earlier than the recorded time of failure. The mean length of the failure sequence under the three failure modes is $2.6\times 10^{5}$, $3.8\times 10^{5}$, and $7.5\times 10^{4}$ samples, respectively [20].

As the utilization of disparate scales for sensor data pertaining to disparate features may prove deleterious to the training of the model, normalization was conducted via the application of Equation (8), thereby ensuring that all sensor values for each recipe were situated within the range [0, 1].

(11) $$x_{i,j}^{\prime }=\frac{x_{i,j}-x_{j\min}}{x_{j\max}-x_{j\min}}$$

In this context, the notation $x_{i,j}$ represents the value of the ith sample with respect to the jth feature, whereas $x_{j\min}$ and $x_{j\max}$ denote the minimum and maximum values observed for the jth feature, respectively.

### 4.2. Model Evaluation Indicators

In this study, the mean squared error (MSE) is calculated using Equation (9) in order to optimize the network parameters.

(12) $$MSE=\frac{1}{m}\sum_{i=1}^{m}{\left(y_{i}-\hat{y_{l}}\right)}^{2}$$

The root mean squared error (RMSE) and the mean absolute error (MAE) are employed to assess the predictive efficacy of the model. The associated formulas are presented in Equations (10) and (11), respectively.

(13) $$RMSE=\sqrt{\frac{1}{m}\sum_{i=1}^{m}{\left(y_{i}-{\hat{y}}_{l}\right)}^{2}}$$

(14) $$MAE=\frac{1}{m}\sum_{i=1}^{m}\left|\left(y_{i}-{\hat{y}}_{l}\right)\right|$$

The mean absolute error (MAE) is a statistical measure that quantifies the discrepancy between the predicted value ${\hat{y}}_{l}$ and the actual value $y_{i}$. The root mean square error (RMSE) is a measure of the discrepancy between the predicted value ${\hat{y}}_{l}$ and the actual value $y_{i}$. A reduction in the MAE and RMSE values of the prediction model will result in enhanced prediction accuracy, which also signifies an elevated RUL prediction accuracy.

### 4.3. RUL Projections

#### 4.3.1. A Comparative Analysis of the Experimental Results

The outcomes yielded by the FECAM-WTCN-Informer model for the F1, F2, and F3 scenarios of the 2018 PHM Data Challenge dataset were contrasted with those of competing models to illustrate the effectiveness and efficiency of the R & D model. These results were quantified by RMSE and MAE metrics, and the results of the F1, F2, and F3 conditions of the proposed model in the 2018 PHM Data Challenge dataset were compared with several competing models, including RNN, LSTM, FNET, TCLSTM, SCINET, DLinear, GRU, and Transformer. This was done in order to test the efficacy and productivity of the proposed model. To evaluate the superiority of the improved WTCN module, experimental comparisons were conducted by replacing the WTCN module in the model with CNN, RES-NET, and TCN, respectively; F-C-I, F-R-I, F-T-I, and F-WT-I in the table represent FECAM-CNN-Informer, FECAM-RESnet-Informer, FECAM-TCN-Informer, and this model, respectively. The superiority of the system can be demonstrated by the presentation of the results, which can be observed in Table 2. In each metric, the smaller the value, the better the result. The optimal results in each metric are shown in bold.

As illustrated in Table 2, FECAM-WTCN-Informer demonstrates superior performance compared to other algorithms in all three specified scenarios. Transformer exhibits robust performance in the two primary scenarios, F1 and F3. However, it exhibits comparatively weaker performance in scenario F2, which involves a limited amount of data. The GRU model performs similarly to the FECAM-WTCN-Informer model, but not as effectively as the proposed model in the F1 scenario with a larger amount of data. The efficiency of the WTCN module was assessed, and the model demonstrated superior performance compared to other replaceable modules, including CNN, RES-NET, and TCN. In conclusion, the FECAM-WTCN-Informer model can be regarded as a reliable solution for addressing long-term time series prediction challenges.

#### 4.3.2. The Results of the Ablation Experiments Are Presented Herewith

In order to evaluate the impact of the individual components in the FECAM-WTCN-Informer, an ablation experiment was conducted in accordance with the proposed methodology. Accordingly, this section delineates six experiments that will be contrasted with the FECAM-WTCN-Informer, all of which were conducted under identical conditions. These experiments utilize various neural network architectures, including a WTCN model, an Informer model, an integrated model combining WTCN and Informer (referred to as WTCN-Informer), an integrated model combining WTCN with the frequency-enhanced channel attention mechanism (FECAM), an integrated model combining Informer with the frequency-enhanced channel attention mechanism (FECAM), a model that combines Informer with the frequency-enhanced channel attention mechanism (FECAM), and a FWT-Informer model that does not use parameter optimization. The objective of these experiments is to facilitate a comparative analysis with the proposed model. The results of these experiments are presented in Table 3.

The optimal values of the results presented in Table 3 are indicated in bold font. It can be observed that the proposed model demonstrates optimal performance in three distinct scenarios. Additionally, the impact of the parameter optimization component is evident when considering the RMSE. In the three specific scenarios, the parameter optimization component enhances the RMSE by 64.05%, 46.41%, and 21.95%, respectively. To evaluate the impact of diverse optimization algorithms on the model, various optimization algorithms (including particle swarm optimization, ant colony, and genetic algorithms) were incorporated into the parameter optimization component for ablation experiments. The experimental outcomes are presented in Table 4.

The optimal values of the results presented in Table 4 are indicated in bold font.The superiority of the SSA algorithm in the parameter optimization component is evident in the mean absolute error (MAE) values. In the F1 scenario, the SSA algorithm exhibits a 77.38%, 63.08%, and 31.22% improvement, respectively, over the other algorithms. Similarly, in the F2 scenario, it demonstrates a 68.70%, 55.83%, and 61.61% improvement, respectively. Furthermore, in the F3 scenario, it exhibits a 69.30%, 8.15%, and 22.27% improvement, respectively.

#### 4.3.3. Calculating Costs and Model Complexity

Table 5 describes the computational cost and complexity of the model. The total number of parameters represents the total number of parameters used in the model training. Training time and inference time show different trends due to the varying sizes of the datasets under the three fault models. The table also shows memory usage and model size.

Table 6 shows a comparison of the training time inference time of our model with the three classical baseline models, and the role of the Informer component in the model can be seen in the F1 mode of operation as an example, where the training time and inference time are much better than those of the LSTM and GRU models, which is not much of an improvement compared to the Transformer model, but it has advantages as well.

#### 4.3.4. Model Visualization

**Figure 9:**
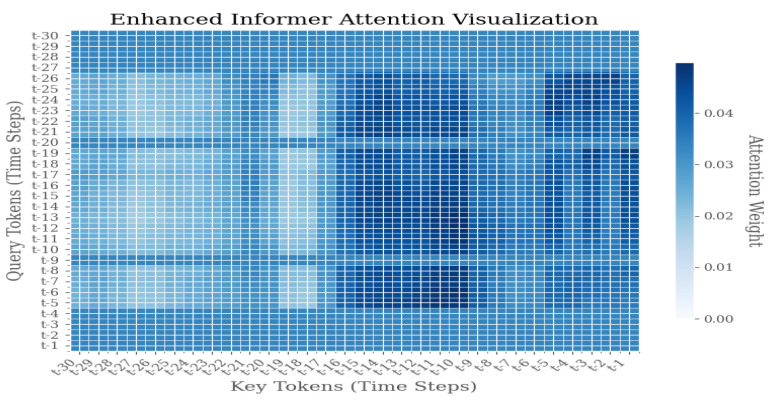
Weight distribution diagram of model visualization under F1 failure mode.

**Figure 10:**
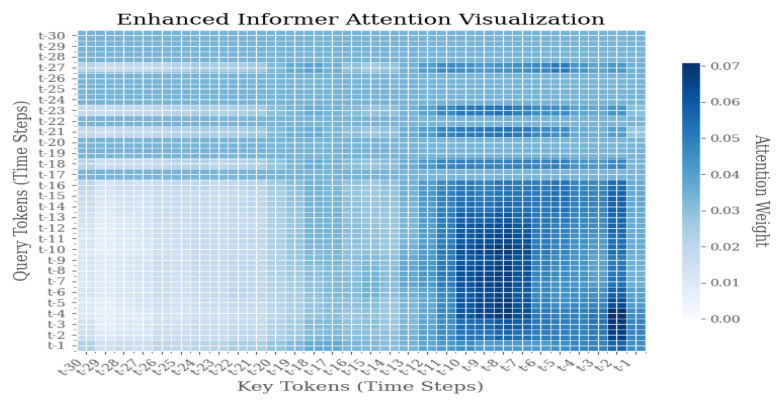
Weight distribution diagram of model visualization under F2 failure mode.

**Figure 11:**
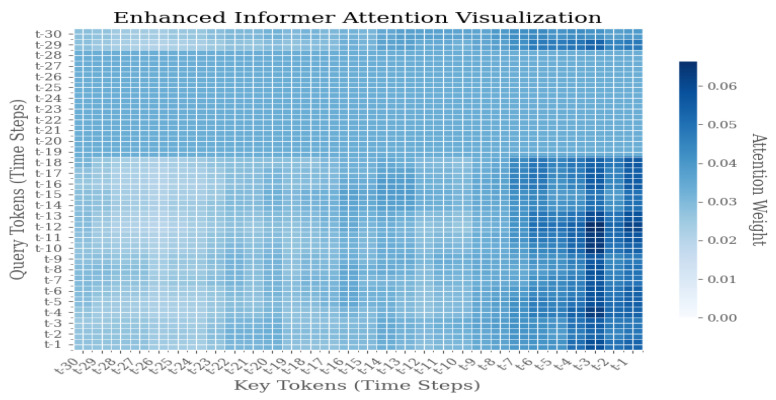
Weight distribution diagram of model visualization under F3 failure mode.

As illustrated in Figure 9, Figure 10 and Figure 11, the Enhanced Informer model demonstrates a distribution of attention weights during the process of time series prediction. The heatmap illustrates the attention weights between query tokens, which represent future time steps from t − 1 to t − 30, and key tokens, which represent historical time steps from t − 30 to t − 1. The color intensity of each pixel is indicative of the magnitude of attention weights, with darker blue signifying higher attention scores.

The attention visualization reveals several key insights into the model’s temporal attention mechanism:

1. Diagonal Pattern: The prominent diagonal pattern indicates that the model places significant emphasis on recent historical information when predicting immediate future steps, thereby aligning with the temporal locality principle in time series forecasting.
2. Sparse Attention Distribution: The sparse attention pattern, characterized by concentrated activity along the diagonal with reduced activity in other regions, suggests that the Enhanced Informer successfully learns to prioritize relevant historical time steps, filtering out extraneous noise and irrelevant information.
3. Temporal Dependency: The attention weights manifest a discernible decay pattern as the temporal distance increases, thereby suggesting that the model appropriately assigns greater weight to recent observations compared to distant historical data.
4. Prediction Horizon Analysis: For prediction horizons extending beyond the immediate term (t − 25 to t − 30), the distribution of attention across multiple historical time steps becomes more pronounced. This phenomenon signifies the model’s capacity to discern long-term dependencies, particularly in scenarios where short-term patterns prove inadequate.

#### 4.3.5. Discussion of Comparisons Between Studies Using the Same Dataset

Research on RUL prediction is already quite mature, but research in semiconductor manufacturing is relatively scarce. In this section, we will compare and discuss our research with that conducted on the same dataset.

Wu et al. [6] proposed multi-layer DW-GRU and DW-GRU-FC models, which improved the life prediction performance of the Flowcool system through sample screening and feature construction, while reducing model complexity and improving computational efficiency. Experimental results show that the GRU model outperforms LSTM in prediction accuracy, and GRU-FCs further improve performance. The data preprocessing methods employed in this study effectively address the issue of excessive redundant data, laying a foundation for future research. In contrast, our model has been evaluated across more algorithms and a broader range of experimental environments, achieving efficient and precise long-term predictions. On the other hand, the TCLSTM model proposed by Hsu et al. [55], which combines attention mechanisms and utilizes TCN for feature extraction and LSTM for capturing temporal dependencies, significantly improves the prediction accuracy of the remaining lifespan of semiconductor equipment. This model can provide reliable maintenance and fault warning information, particularly suitable for ion etching equipment. By stabilizing input data through data preprocessing, prediction errors are reduced, and deficiencies in temporal feature extraction are improved. Similarly, Ahmed et al. [56] adopted a model combining TCN, LSTM, and self-attention mechanisms, but it lacked innovation, and the prediction accuracy did not show significant improvement.

In summary, we can see the advantages of TCN and self-attention mechanisms. Inspired by reasonable data processing methods, we have improved the TCN model, selected a more effective attention mechanism, and used the highly efficient Informer component, which is good at predicting long-term sequence data while also offering impressive efficiency.

#### 4.3.6. Model Predictions

**Figure 12:**
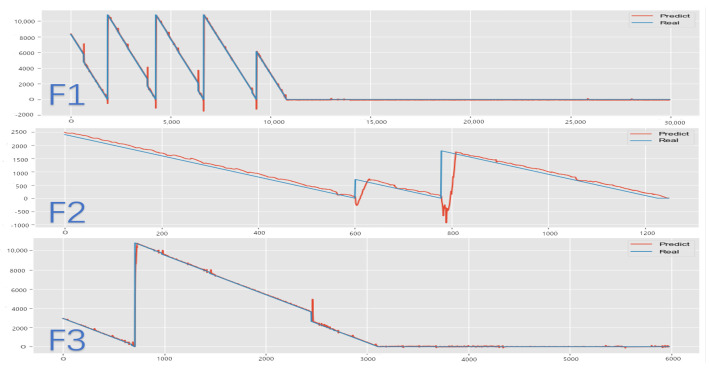
Comparison of prediction results before parameter optimization.

**Figure 13:**
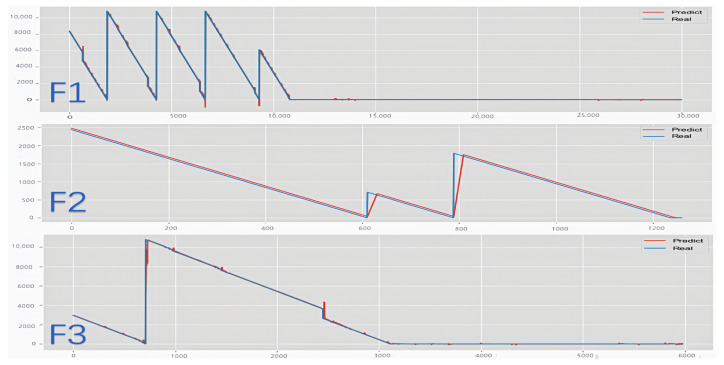
Comparison chart of forecast results.

This subsection illustrates the forecasting of remaining useful life (RUL) by the FECAM-WTCN-Informer model under three distinct scenarios for the test set (01_M02_DC_test) in the Flowcool dataset, as depicted in Figure 12 and Figure 13. The two columns represent images before and after parameter optimization under the aforementioned scenarios, respectively. The results demonstrate that the parameter optimization component enhances the smoothing of the prediction curves and reduces the burr of prediction at the inflection points in the data. The model presented in this paper is ultimately capable of achieving precise results in prediction, thereby demonstrating the efficacy of this model in addressing the challenge of forecasting the remaining operational lifespan of an ion etcher’s cooling system.

## 5. Discussion

This study introduces a novel method, FECAM-WTCN-Informer, for predicting the remaining useful life (RUL) during ion mill etching. The FECAM-WTCN-Informer model integrates the WTCN, Informer, and the frequency-enhanced channel attention mechanism. The enhanced TCN is employed to capture extended dependencies in the time series data. While the global sensing field is enhanced, FECAM is responsible for frequency domain feature extraction and enhances key feature selection. The processed features are then input to the Informer network for RUL prediction. The experimental evaluation is conducted using the PHM 2018 Challenge dataset. A comparative analysis with the leading models in the field demonstrates that the proposed scheme effectively addresses the issues of excessive irrelevant data and suboptimal prediction, particularly for long-term data prediction, and outperforms the existing methods in this domain. The model achieves a model fit of 99.82%, 98.65%, and 99.57% in F1, F2, and F3 modes, respectively, with RMSE indices of 96.72%, 5.41%, and 46.86% lower than the best prediction models for current flow-cooling systems and MAE indices of 91.85%, 55.61%, and 48.98% lower than the best prediction models for current flow-cooling systems. Furthermore, the tests on real data illustrate the effectiveness of the model.

However, it is imperative to acknowledge the inherent limitations of this work. The ion mill etching process is not universally applicable; the model is contingent on the preprocessing of the data. To ensure the validity of the training data, the data from other industrial systems during the aforementioned process is not applicable. The hyperparameters were optimized through further model refinement, a process that is not efficient.

In conclusion, the research on remaining useful life (RUL) prediction is an evolving topic. The model can be enhanced in a number of ways. For example, rather than focusing exclusively on the ion mill etching process, it would be advantageous to incorporate additional relevant sub-processes in the semiconductor production process. Furthermore, incorporating the prediction of multi-machine scenarios would enhance the practical applications of the model. The Informer component of the model is used to address long-term sequences, and as a final prediction component in the model, it can be further optimized by, for example, introducing iterative corrections. The utilization of mechanisms such as reinforcement learning, meta-learning, and related approaches has been demonstrated to enhance the precision and computational efficiency of Informer. The implementation of more advanced variants of Transformer has also been proposed as a potential replacement for this component. Furthermore, the incorporation of interpretable AI components has been identified as a promising avenue for future enhancements.

In addition to this, this research is important to the deployment of online learning models. Additional experiments are needed to explore the feasibility of this aspect, and future work can be based on the pipeline latency required by the industry for response latency evaluation as well as streaming data and for the quality of the data, such as missing data estimation strategies and graceful degradation strategies in the case of multi-sensor failures. Appropriate online learning strategies are the most important. In conclusion, our model is challenging to integrate in the current industrial industry, due to the shortage and imbalance of datasets; the model performs well but still has many shortcomings. Hopefully, it will be improved in the future.

## Figures and Tables

**Table 1:** 2018 PHM Data Challenge dataset sensors description.

Name	Description
Time	time
Tool	tool id
Stage	processing stage of the wafer
Lot	wafer id
Run num	number of times the tool has been run
Recipe	tool settings used to process the wafer
Recipe step	the process step of a recipe
iongaugepressure	pressure reading for the main process chamber when k is under vacuum
etchbeamvoltage	the voltage potential applied to the beam plate of the grid assembly
etchbeamcurrent	ion current impacting the beam grid determining the amount of ions accelerated through the grid assembly to the wafer
etchsuppressorvoltage	voltage potential applied to the suppressor plate of the grid assembly
etchsuppressorcurrent	ion current impacting the suppressor grid plate
flowcoolflowrate	rate of flow of helium through the flow-cool circuit, controlled by the mass flow controller
flowcoolpressure	resulting helium pressure in the flow-cool circuit
etchgaschannel1readback	rate of flow of argon into the source assembly in the vacuum chamber
etchpbngasreadback	rate of flow of argon into the PBN assembly in the chamber
fixturetiltangle	wafer tilt angle setting
rotationspeed	wafer rotation speed setting
actualrotationangle	measure of the wafer rotation angle
fixtureshutterposition	open/close shutter setting for the wafer shielding
etchsourceusage	counter of use for the grid assembly consumable
etchauxsourcetimer	counter of use for the chamber shields consumable
etchaux2sourcetimer	counter of use for the chamber shields consumable
actualstepduration	measured time duration for a particular step

**Table 2:** Model comparison experimental results.

	F1		F2		F3	
NORM	RMSE	MAE	RMSE	MAE	RMSE	MAE
RNN	1488.8	1009.3	1079.1	858.49	2850.29	1921.7
LSTM	837.33	223.44	798.88	273.17	549.88	157.63
FNet	896.58	218.11	2393.02	1144.40	1241.67	406.96
TCLSTM	774.04	220.64	794.99	263.07	544.61	123.92
SCINET	821.84	187.64	2417.05	479.05	1320.10	371.48
DLinear	684.89	73.26	3089.50	1596.82	1052.14	290.40
GRU	506.64	121.73	145.16	59.77	329.62	178.14
Transformer	392.28	98.60	325.79	280.93	403.33	73.34
F-C-I	317.69	34.85	228.04	80.22	296.43	179.63
F-R-I	235.88	70.96	185.55	58.78	258.64	158.36
F-T-I	41.68	38.89	176.23	64.36	211.81	134.05
F-WT-I	**12.86**	**5.97**	**137.31**	**26.53**	**175.17**	**37.42**

**Table 3:** Model comparison experimental results.

	F1		F2		F3	
NORM	RMSE	MAE	RMSE	MAE	RMSE	MAE
WTCN	736.41	225.18	758.73	211.84	754.77	263.7
Informer	501.79	300.48	612.85	468.11	391.51	332.9
WTCN-Informer	581.62	98.14	266.76	120.42	314.66	61.56
F-WTCN	270.93	135.59	211.92	79.15	318.89	72.69
F-Informer	219.13	93.56	265.36	84.43	271.40	66.58
FWT-Informer	35.77	33.24	256.19	104.45	234.52	58.39
Proposed model	**12.86**	**5.97**	**137.31**	**26.53**	**175.17**	**37.42**

**Table 4:** Experimental results comparing different optimization algorithms.

	F1		F2		F3	
NORM	RMSE	MAE	RMSE	MAE	RMSE	MAE
Unoptimized	35.77	33.24	256.19	104.45	234.52	58.39
PSO	30.13	26.39	250.04	84.77	224.44	121.89
ACO	23.51	16.17	227.59	60.07	220.61	40.74
GA	14.53	8.68	245.40	69.11	215.41	48.14
SSA	**12.86**	**5.97**	**137.31**	**26.53**	**175.17**	**37.42**

**Table 5:** Model cost and complexity.

	F1	F2	F3
Total parameters	154,065	154,065	154,065
Training time/s	2846.50	641.47	2590.76
Average inference time (1 sample)/ms	12.8570	11.2660	11.5056
Max GPU memory used/MB	8053.63	526.19	2428.53
Memory Usage/MB	2121.86	1646.72	1875.83
Saved model size/MB	3.06	3.06	3.06

**Table 6:** Comparison table of training reasoning time.

	Our Model	GRU	Transformer	LSTM
Training time/s	1666.77	5040	3122.37	4860
Average inference time (1 sample)/ms	0.0235	0.1148	0.0449	0.1136

## Data Availability

All data in the form of graphs are contained within the article. The raw data are available on request to the corresponding author.
